# Role of miR-34a as a suppressor of L1CAM in endometrial carcinoma

**DOI:** 10.18632/oncotarget.1552

**Published:** 2014-01-12

**Authors:** Uwe Schirmer, Kai Doberstein, Anne-Kathleen Rupp, Niko P. Bretz, Daniela Wuttig, Helena Kiefel, Christian Breunig, Heidi Fiegl, Elisabeth Müller-Holzner, Robert Zeillinger, Heidi Eva, Alain G. Zeimet, Holger SÜltmann, Peter Altevogt

**Affiliations:** ^1^ Department of Translational Immunology, German Cancer Research Center; ^2^ Working Group Cancer Genome Research, German Cancer Research Center; ^3^ Department of Gynecology and Obstetrics, Medical University of Innsbruck, A-6020 Innsbruck, Austria; ^4^ Medical University Vienna, Vienna, Austria; ^5^ Division of Molecular Genome Analysis, German Cancer Research Center, Heidelberg, Germany

**Keywords:** miR-34a, endometrial carcinoma, L1CAM, ovarian carcinoma

## Abstract

L1CAM promotes cell motility, invasion and metastasis formation in various human cancers and can be considered as a driver of tumor progression. Knowledge about genetic processes leading to the presence of L1CAM in cancers is of considerable importance. Experimentally, L1CAM expression can be achieved by various means. Overexpression of the transcription factor SLUG or treatment of cells with TGF-ß1 can induce or augment L1CAM levels in cancer cells. Likewise, hypomethylation of the L1CAM promoter on the X chromosome correlates with L1CAM expression. However, presently no mechanisms that might control transcriptional activity are known. Here we have identified miR-34a as a suppressor of L1CAM. We observed that L1CAM positive endometrial carcinoma (EC) cell lines HEC1B and SPAC1L lost L1CAM protein and mRNA by treatment with demethylating agents or knock-down of the DNA-methyltransferase-1 (DNMT1). Concomitantly, several miRNAs were up-regulated. Using miRNA profiling, luciferase reporter assays and mutagenesis, we identified miR-34a as a putative binder to the L1CAM-3'UTR. Overexpression of miR-34a in HEC1B cells blocked L1CAM expression and inhibited cell migration. In ECC1 cells (wildtype p53) the activation of p53 caused miR-34a up-regulation and loss of L1CAM expression that was miR-34a dependent. We observed an inverse correlation between L1CAM and miR-34a levels in EC cell lines. In primary tumor sections areas expressing high amounts of L1CAM had less miR-34a expression than those with low L1CAM levels. Our data suggest that miR-34a can regulate L1CAM expression by targeting L1CAM mRNA for degradation. These findings shed new light on the complex regulation of L1CAM in human tumors.

## INTRODUCTION

Tumor cells in vivo have a high phenotypic plasticity that is likely to be required to survive and escape the host immune system. A hallmark of cancer progression is the acquisition of invasion and metastasis capacity [1, 2]. There is increasing evidence that interactions between the primary tumor and the ambient stroma creates a microenvironment that enables single tumor cells to metastasize [2]. During metastasis, tumor cells at the invasive front detach from the tumor mass and invade the surrounding tissue. Metastatic tumor cells often undergo EMT, a process characterized by up-regulation of motility promoting molecules such as the adhesion molecule L1CAM and the loss of epithelial markers such as E-cadherin and cytokeratines [3, 4]. At the same time the activation of the EMT inducing TFs SNAIL, SLUG, or Twist is observed [5, 6].

L1CAM plays an important role in the development of the nervous system and in the malignancy of human tumors [7]. L1CAM is overexpressed in many human carcinomas and augments cell motility, invasion and metastasis formation [8]. Several studies have shown that L1CAM positive carcinomas have a bad prognosis [7, 9]. For ECs we reported before that type II tumors, representing the most aggressive serous and clear-cell EC are positive for L1CAM [10]. In addition, a recent study has shown that also the less aggressive endometrioid EC (type I tumors) can occasionally express L1CAM conferring a bad prognosis to those patients [11].

The expression of L1CAM in tumor cells can be enhanced by various means. For ECs we demonstrated that L1CAM is up-regulated by TGF-ß treatment in an EMT-like fashion or by overexpression of the transcription factor SLUG [3, 10]. Similar observations were made for pancreatic cancer [12]. Interestingly, recent reports have shown that in cancer cell lines the L1CAM expression is augmented by treatment with DNA-demethylation agents that affect the methylation status of the L1CAM promoter [13–15]. Thus, existing data suggest that up-regulation of L1CAM at the transcriptional level can be achieved by various mechanisms. However, little is known about molecular mechanisms that suppress the expression of L1CAM in cancers.

MicroRNAs (miRNAs) are 22 nucleotide non-coding RNAs that are powerful regulators of gene expression. Dysregulation of miRNAs in cancer cells are known to be important in different steps of the tumorgenesis, from initiation and development to progression toward the acquisition of a metastatic phenotype [16, 17]. MicroRNAs can bind to specific binding sites in gene transcripts often located in the 3'UTR and thereby target them to degradation by the RISC complex.

Here we have identified miR-34a as a suppressor of L1CAM in EC cells. Surprisingly, we observed that 5′-AzaC treatment of L1CAM positive EC cells led to rapid down-regulation of L1CAM that was accompanied by up-regulation of various miRNAs. Using L1CAM 3′-UTR specific assays, mutational analysis and overexpression we demonstrate that miR-34a is responsible for L1CAM down-regulation. Our results shed new light on L1CAM regulation in tumors and show for the first time a link between the p53/miR-34a axis and L1CAM in ECs.

## RESULTS

### 5′-AzaC treatment differentially affects expression of L1CAM

**Figure 1:**
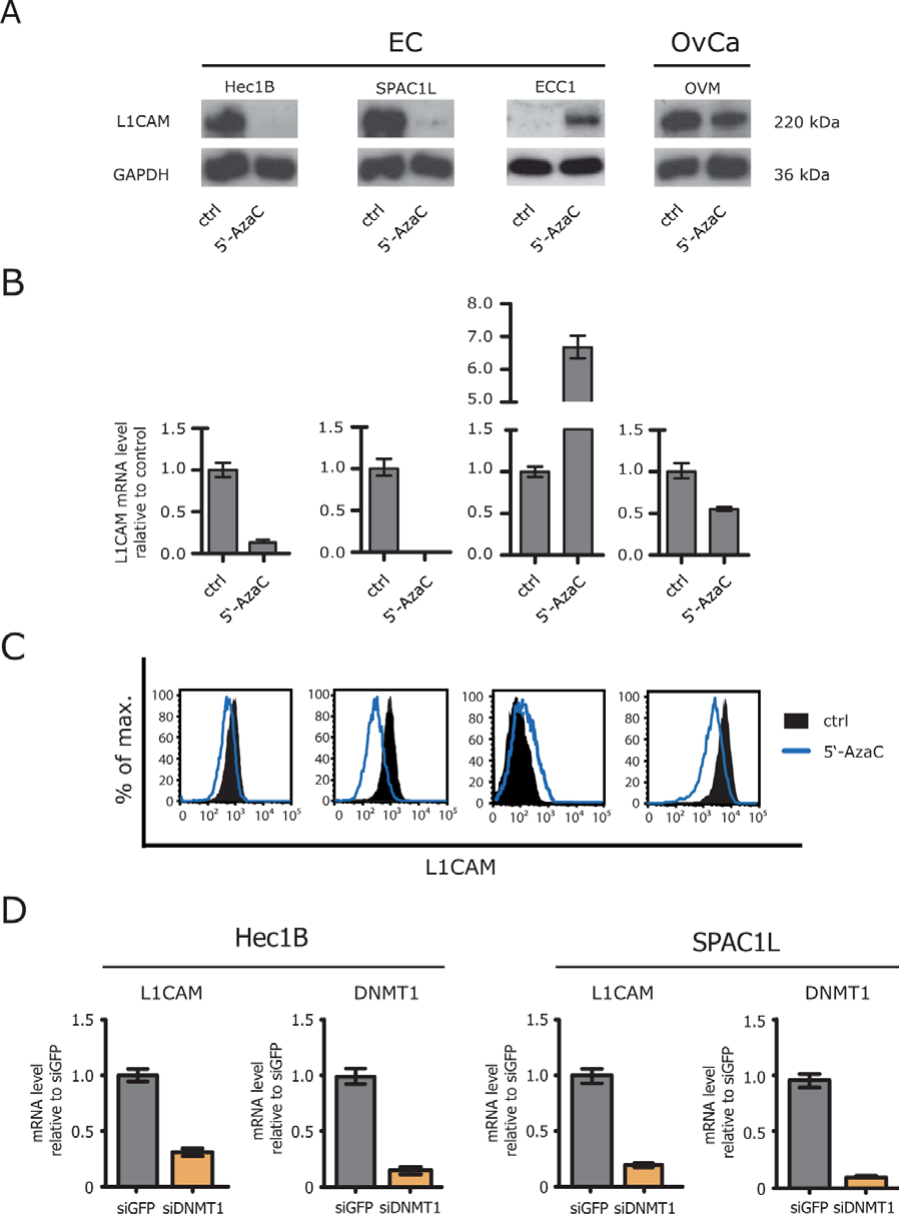
Loss of L1CAM expression by demethylating agents (A) Cells were treated for 5 days with the indicated concentration of 5′-AzaC. Normal medium was used as a mock control. Cell lysates were prepared for Western blot analysis. MAb L1-11A was used as a primary antibody followed by peroxidase conjugated Goat anti mouse IgG and ECL detection. (B) Cells were lysed and mRNA was isolated and transcribed into cDNA. RT-PCR analysis was performed using ß-actin as internal standard. (C) Cells were treated with 5′-AzaC as described above and subjected to cytofluorographic analysis. (D) DNMT1 was depleted by specific siRNA and the level of L1CAM expression were analysed by RT-PCR. The knock-down efficiency for DNMT1 is also shown.

We reported before, that the L1CAM expression in EC cell lines is induced or augmented by treatment with the DNA-demethylating agent 5′-AzaC [15]. In line with this, a strong up-regulation of L1CAM at the mRNA and protein level was observed in ECC1 cells that are only weakly positive for L1CAM (Fig. 1A and B). In strong contrast, a similar treatment of the L1CAM positive EC cell lines HEC1B and SPAC1L lead to a loss of L1CAM protein and mRNA (Fig. 1A and B). In OVMz ovarian carcinoma cells the level of L1CAM was also clearly reduced upon treatment. These findings were corroborated by FACS analysis revealing a clear reduction of L1CAM cell surface expression.

(Fig. 1C). Similar results were obtained when Decitabine instead of 5′-AzaC was used (data not shown).

To rule out the possibility that the pharmacological inhibition caused unspecific side effects, we depleted DNMT1 by specific siRNA. The DNMT1 knockdown significantly reduced the level of L1CAM mRNA in HEC1B as well as SPAC1L (Fig. 1D). These findings suggested that the interference with DNA-demethylation can differentially affects the expression of L1CAM.

### HDAC inhibitor TSA does not suppress L1CAM

**Figure 2:**
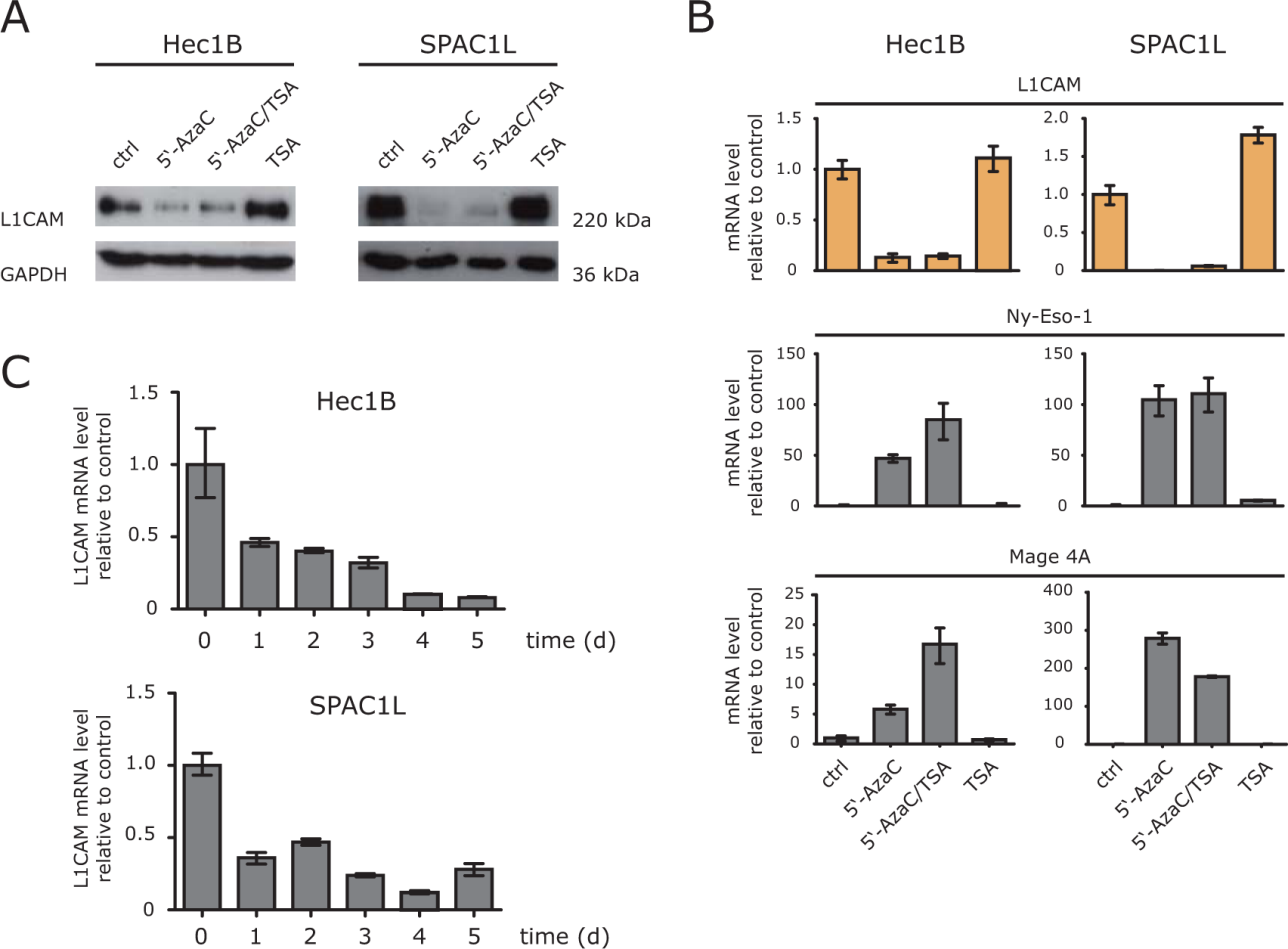
HD AC inhibitors fail to induce L1CAM down-regulation (A) The indicated cell lines were treated with 5-AzaC, 5′-AzaC/TSA or TSA for 5 days and the cell lysates were used for western blot analysis with specific antibodies against L1CAM and GAPDH. (B) The expression levels of L1CAM and the cancer testis antigens Ny-Eso-1 and MAGE-A4 were analysed by RT-PCR in cells treated as described above. (C) Time kinetic of L1CAM down-regulation. Cells were treated for the indicated length of time with 5′-AzaC. Normal medium was used as a mock control. Analysis of LI CAM expression was performed by RT-PCR using ß-actin as internal standard.

In L1CAM low/negative cells it has been shown, that the treatment with HDAC inhibitors up-regulates L1CAM expression [13, 15]. We therefore examined whether TSA treatment would also lead to down-regulation in L1CAM positive cells. Clearly, L1CAM suppression was only observed in cells treated with 5′-AzaC or in combination with TSA but not with TSA alone (Fig. 2A). Importantly, the DNA-methylation sensitive genes *MAGE-A4* and *Ny-Eso-1* showed the expected up-regulation (Fig. 2B).

Additional kinetic experiments showed that the loss of L1CAM proceeded in a time-dependent fashion (Fig. 2C). We concluded that in L1CAM positive cells 5′-AzaC but not TSA induced a strong and specific suppressive effect on L1CAM expression.

### miRNA profiling identifies miR-34a as potential regulator

**Figure 3:**
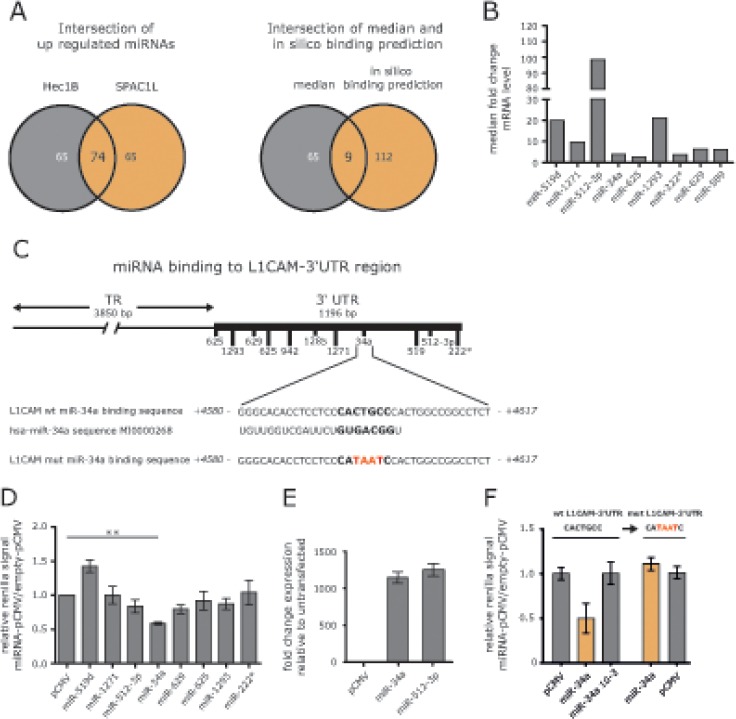
Identification of miRNAs involved in L1CAM regulation (A) HEC1B and SPAC1L cells were treated or non-treated with 5′-AzaC and subjected to miRNA profiling. Regulated miRNAs were compared between both cell lines. Common miRNAs were subjected to bioinformatic s analysis for their ability in silico to bind to the L1CAM 3′-UTR region. (B) Median fold changes of the 9 selected miRNAs are shown. (C) Lhe L1CAM-3′ UTR comprises 1196 bp and putative miRNA binding sites are indicated. Lhe miR-34a binding site is shown in large and the hsa-miR-34a sequence is also indicated. (D) Lhe indicated miRNAs were cloned into in pCMV-MIR and co-transfected with a L1CAM-3′ULR reporter plasmid into HEC1B cells. Cells were lysed and luciferase activity was measured after 48 h. Data are given as quotient of empty vector versus L1CAM-3 ′UTR reporter vector. (E) Representative values for miRNA overexpression are shown for miR-34a and miR512-3p. (F) Lhe miR-34a binding site in the L1CAM-3′ULR reporter vector was changed by site directed mutagenesis and the sequence was confirmed by DNA-sequencing (mutL1CAM-3 ′UTR). Likewise, a mutant form of miR-34a devoid of the seed sequence was generated. Wildtype and mutated L1CAM-3′ ULR plasmids were co-transfected with miR-34a or diluted miR-34a (10^−3^) or empty vector into HEC1B cells. Cells were lysed and luciferase activity was measured after 48 h.

5′-AzaC treatment of cells is known to affect the activity of many genes including those encoding miRNAs. We postulated that the up-regulation of particular miRNAs might be responsible for the reduced expression of L1CAM. Therefore we carried out a miRNA profiling by comparing non-treated to 5′-AzaC-treated HEC1B and SPAC1L cells. We identified 74 miRNAs that were co-regulated in both cell lines (Fig. 3A).

In addition, we used bioinformatic data on putative miRNA binding sites in the 3′-UTR region of the L1CAM gene depicted in Fig. 3B. Applying these tools, we identified 9 miRNAs up-regulated in both cell lines (Fig.3A). Strongest regulation was observed for miR-519d, miR-512-3p and miR-1293 (Fig. 3C).

### miR-34a targets the 3′UTR sequences of L1CAM

To verify which miRNA might have regulatory capacity for L1CAM, we cloned the genomic sequences of the identified miRNAs into pCMV-MIR. We performed reporter assays in HEC1B cells by co-transfecting the cloned miRNAs together with a L1CAM-3′UTR reporter plasmid. Each analysis was done in comparison to the empty reporter plasmid and the results are summarized in Fig. 3D. Overexpression of pCMV-miR-34a showed the strongest reduction of reporter activity (Fig.3D and E). Mutagenesis of the miR-34a binding site in the 3′UTR reporter construct or in the miR-34a seed region (see Fig. 3B) abolished the suppressive effect in the reporter assay (Fig.3F).

### Overexpression of miR-34a affects L1CAM expression

**Figure 4:**
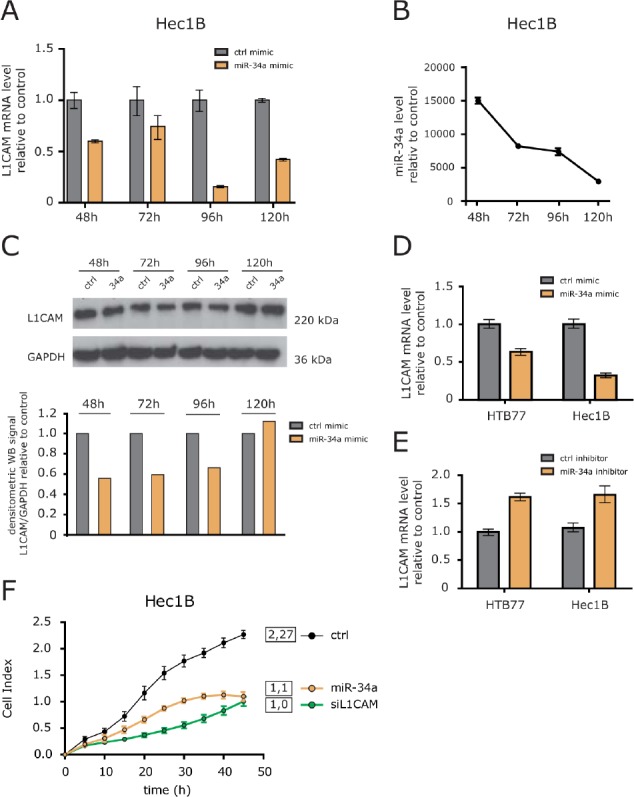
Identification of miRNAs involved in L1CAM regulation (A) L1CAM expression levels after overexpression of miR-34a were determined by RT-PCR analysis at the indicated time points. Results are compared to a control miRNA (ctr1-mimic). (B) Efficiency of miR-34a overexpression was tested in parallel. (C) L1CAM protein expression in cells overexpressing miR-34a. Cells were lysed at the indicated time points and subjected to Western blot analysis. Densitometrical analysis of the gel bands for L1CAM are given. (D) miR-34a mimic and (E) miR-34a-inhibitor were overexpressed in HEC1B and HTB77 ovarian carcinoma cells. L1CAM expression levels were determined by RT-PCR after 96 h. (F) HEC1B cells overexpressing miR-34a were subjected to cell migration analysis.

To verify whether miR-34a was able to regulate L1CAM, we overexpressed miR-34a encoding oligonucleotides in HEC1B cells. Transfection efficacy was verified by RT-PCR analysis. By comparing miR-34a versus a control oligonucleotide, a time-dependent decrease of L1CAM mRNA was observed that peaked at 96 h after transfection (Fig. 4A). L1CAM protein levels were likewise affected by miR-34a although to a lesser extent (Fig. 4B).

We next tested whether inhibition of endogenous miR-34a would affect L1CAM expression levels in HEC1B and HTB77 cells. Whereas overexpression of miR-34a clearly decreased L1CAM expression as expected, the miR-34a inhibitor increased it in both cell lines (Fig.4C). These results confirmed that miR-34a acts as a regulator of L1CAM levels in tumor cell lines.

L1CAM can profoundly affect cell migration and invasion of tumor cells [8]. To test whether overexpression of miR-34a accompanied with L1CAM loss had similar effects, we investigated cell migration after miR-34a transfection. We observed a clearly reduced cell migration that was comparable in magnitude to the depletion of L1CAM by specific siRNA (Fig.4D). In summary, these results illustrate that the overexpression of miR-34a can suppress the migratory capacity of tumor cells.

### Treatment of ECC1 cells with Nutlin-3a blocks L1CAM expression

**Figure 5:**
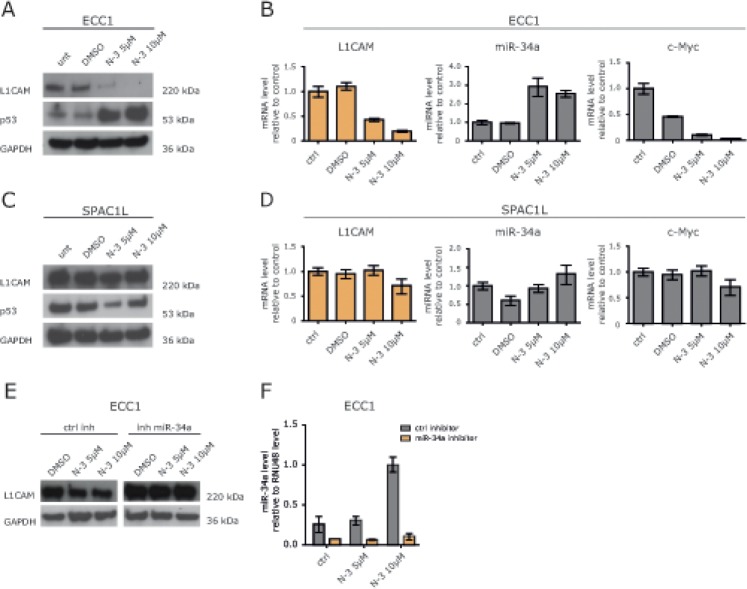
Nutlin-3a down-regulates L1CAM in wildtype p53 ECC1 cells (A) ECC1 cells were treated with the indicated concentration of Nutlin-3a for 48 h. DMSO served as solvent control. Cells were lysed and subjected to Western blot analysis. Antibodies against L1CAM, p53 and GAPDH were used as primary antibodies followed by peroxidase conjugated second antibodies and ECL detection. (B) L1CAM and miR-34a expression levels after Nutlin-3a treatment were determined by RT-PCR. c-Myc regulation served as positive control for Nutlin-3a effectivity. (C+D) SPAC1L cells carrying a p53 mutation were treated with Nutlin-3a and analysed as described before. (E) Western blot analysis of ECC1 cells after miR-34a inhibitor transfection and Nutlin-3a treatment. Cells were transfected with miR-34a inhibitor or control oligonucleotides and after 24h of incubation subsequently treated with Nutlin-3a for another 48h. (F) miR-34a expression after inhibitor transfection and Nutlin-3a treatment. The experiment was done as described before and the miR-34a levels were determined by stem loop qPCR.

There is evidence that p53 regulates a miRNA network including the miR-34 and miR-200 family [18]. ECC1 cells have a low expression level for L1CAM (Fig.5A) and possess a p53 wildtype genotype. Next, we tested whether the induction of endogenous miR-34a would influence L1CAM expression. Therefore, ECC1 cells were treated with Nutlin-3a, a small molecule inhibitor of MDM2 that has been shown to activate p53 [19] leading to the transcription of the miR-34 genes [20], Indeed, Nutlin-3a treatment up-regulated p53 and miR-34a expression in a dose-dependent manner (Fig. 5A and B). It also blocked expression of L1CAM at the mRNA and protein level (Fig.5A and B). As expected, *c-Myc*, a known target gene of miR-34a, was down-regulated as well. (Fig.5B).

We also investigated the effects of Nutlin-3a treatment in SPAC1L cells that carry a mutation of p53 (Pos. 273, Arg to His). In these cells Nutlin-3a was ineffective and neither down-regulation of L1CAM nor up-regulation of miR-34a occurred (Fig.5C and D). Likewise, *c-Myc* was not regulated (Fig.5E). Similar results were obtained in HEC1B cells that carry another mutation in p53 (Pos. 248, Arg to G1n) (data not shown).

To demonstrate that the down-regulation of L1CAM in ECC1 cells was mediated by miR-34a, we transfected ECC1 cells with the miR-34a inhibitor or the control inhibitor and treated the cells 24 h later with Nutlin-3a. As shown in Fig. 5E and F, the miR-34a inhibitor but not the control inhibitor attenuated the Nutlin-3a induced down-regulation of L1CAM. These results provided evidence that miR-34a was responsible for the L1CAM loss and suggested that only an intact p53/miR-34a axis can suppress L1CAM.

### Inverse correlation between levels of miR-34a and L1CAM expression

**Figure 6:**
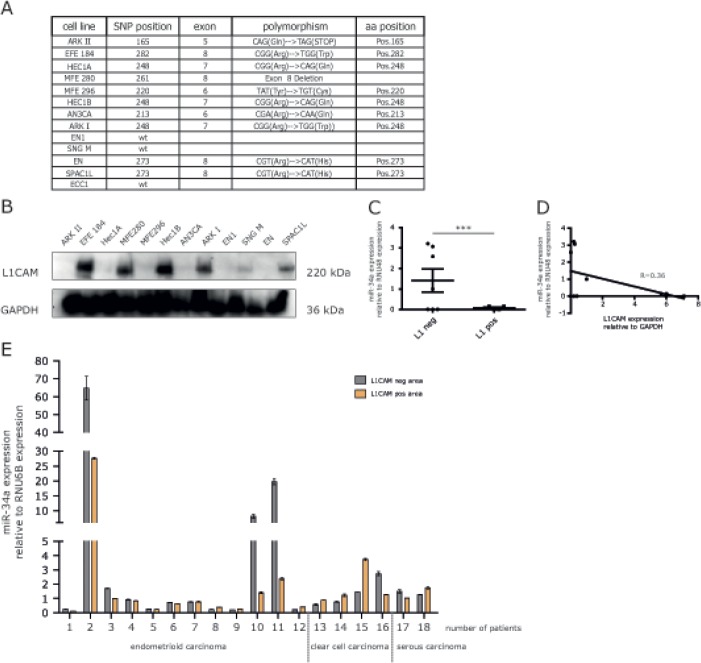
Expression of miR-34a and L1CAM are inversely correlated in EC cell lines and cancer tissues (A) Mutation status of p53 in different endometrial carcinoma cell lines. Indicated are the positions as well as sequences of the polymorphisms. (B) Western blot analysis of L1CAM expression in a panel of EC cell lines. Cells were lysed and subjected to Western blot analysis using mAb L1-11A as a primary antibody followed by peroxidase conjugated Goat anti mouse IgG and ECL detection. (C) Scatter blot of miR-34a expression in L1CAM positive (MFE280, HEC1B, ARK I, SPAC1L) and L1CAM negative (ARK II, Hec1A, MFE296, AN3CA, ENI, SNGM) cell lines (D) Correlation analysis of the relative miR-34a and L1CAM expression in a panel of EC cell lines. (E) Comparison of the relative miR-34a expression in L1CAM positive and negative areas of EC tumor sections from 18 patients. Patient samples were subdivided according to their different tumor phenotypes.

To assess a role of endogenous miR-34a in the regulation of L1CAM, a panel of EC cell lines was studied for their expression of L1CAM and the mutational status of p53 (Fig.6A). Several cell lines showed significant expression of L1CAM as detected by Western blot analysis (Fig.6B). We observed an inverse correlation between L1CAM and miR-34a expression levels (Fig.6C). To extend these findings we examined L1CAM and miR-34a in primary ECs. RNA was extracted from both L1CAM positive or negative tumor areas as described before [15]. The results from paired areas of the same tumors are summarized in Fig. 6D and show that high miR-34a expression was only detected in 3/18 analyzed tumors. In these cases reduced levels of miR-34a were observed in L1CAM positive areas (Fig.6D). However, due to the small number of cases these findings did not reach statistical significance.

## DISCUSSION

The control of L1CAM expression in cancers appears to be complex and is subject to both transcriptional and epigenetic regulation. Here we demonstrate that i) treatment of L1CAM positive EC cells with DNA demethylating agents lead to rapid loss of L1CAM protein and mRNA expression; ii) miR-34a is a putative binderte the L1CAM -3′UTR; iii) manipulation of miR-34a levels can affect L1CAM expression levels; iv) an inverse correlation between the L1CAM and miR-34a expression was noted in EC cell lines and primary tumor tissues. Collectively, our results show for the first time that L1CAM is regulated by miR-34a.

We among others have previously reported that the treatment of cells with demethylating agents can induce or augment the expression of L1CAM in otherwise low or negative cells [14, 15]. Importantly, using similar conditions for treatment we observed that in L1CAM strongly positive cells the exact opposite happened. In HEC1B and SPAC1L EC cells the L1CAM expression was lost at the protein and mRNA level. This contrasting outcome is unlikely due to changes in the methylation of the L1CAM promoter. We have shown before that in SPAC1L and HEC1B cells the promoter is hypomethylated [15] and further analysis revealed that 5′-AzaC treatment did not alter this status (H.Fiegl unpublished observations). We took this as an indication that the treatment had activated a genetic control mechanisms previously silenced in these cells. We searched for miRNAs that are known to target specific mRNAs for its degradation and identified miR-34a as a putative binder to the L1CAM-3′UTR region. MiR-34a is known as a tumor suppressor miRNA and is a transcriptional target of p53 [21]. MiR-34a was shown to play an important role in the regulation of EMT [22] and can target a variety of genes involved in tumor progression such as c-Met [23], SNAIL [24], E2F3a [25], PDGFR-aß [26]. Most recently, reduced chemoresistance and migration, as well as regulation of sternness markers in colorectal cancer cells were associated with miR-34a mediated repression of c-Kit by p53. [27].

We observed that overexpression of miR-34a down-regulated L1CAM at the mRNA and protein level in HEC1B cells and HTB77 cells. In line with this, a miR-34a inhibitor was able to boost L1CAM expression. Due to intrinsic difficulties to achieve high transfection efficacy, similar experiments could not be done in SPAC1L. We also observed that miR-34a overexpression lead to a significant decrease in cell motility. At this stage it is unclear whether we are solely targeting L1CAM-dependent motility as miR-34a can also interfere with c-Met-driven invasion and cell motility [23].

Another way to increase miR-34a levels is by activating p53. In fact, using p53-wildtype expressing ECC1 cells, we observed an up-regulation of miR-34a that was paralleled by a loss of L1CAM expression. Using the miR-34a inhibitor to block newly produced miR-34a this effect was attenuated suggesting that L1CAM down-regulation was indeed miR-34a-dependent. Importantly, Nutlin-3a was unable to induce these effects in mutant p53 expressing SPAC1L and HEC1B cells. These data suggested that only an intact p53-miR-34a axis can suppress L1CAM expression.

EMT is a key event during the onset of metastasis and miR-34a is involved in EMT by regulation of the TF SNAIL [24]. Interestingly, we reported before that overexpression of SLUG, that is related to SNAIL, can augment L1CAM expression and a correlation exists between L1CAM and SLUG expression levels [15]. It was therefore of interest to analyze whether elevated miR-34a levels might reduce SNAIL/SLUG expression and inhibit L1CAM expression indirectly by preventing transcription of the L1CAM gene. We failed to detect significant changes in SNAIL/SLUG levels at the RNA level in HEC1B cells after overexpression of miR-34a. Likewise, treatment of ECC1 cells with Nutlin-3a augmented SLUG/SNAIL levels rather than decreased them (RA. unpublished results). We conclude that the TFs SNAIL/SLUG do not significantly contribute to the observed down-regulation of L1CAM in EC cells.

A certain limitation of our study is that we do not know whether the cell lines we used in our study reflect the clinical situation of ECs. It is quite known that EC can be divided on the molecular level into two subtypes. Type I ECs are associated with PTEN inactivation and express ER/PR whereas in type II ECs the ER/PR expression is usually lost and p53 mutations are present [28]. A recent integrated genomic characterization of ECs has led to a more refined classification [29]. In previous work we have shown that type II ECs are generally positive for L1CAM, a feature shared by approx. 50% of the EC cell lines under study in the present report. Most of these cell lines also harboured p53 mutations but we did not discover a correlation between mutant p53 and L1CAM expression. Instead, we observed an inverse correlation between miR-34a and L1CAM in our cell lines. In EC tumor tissues we found elevated miR-34a levels only in 3/18 endometrioid ECs that are considered type I. In these cases we noted that L1CAM negative areas expressed higher amounts of miR-34a than the respective L1CAM positive areas. In the 6 samples derived from clear cell and serous EC (type II) the miR-34a levels were generally very low suggesting that these tumors had lost miR-34a expression.

Collectively, our results *in vivo* and *in vitro* provide evidence for a role of miR-34a in the regulation of L1CAM. These data shed new light on the complexity of mechanisms regulating the expression of L1CAM in human tumors.

## METHODS

### Cells, chemicals and antibodies

The endometrial carcinoma cell line ECC1 was maintained in DMEM/F12 medium. HEC1B, SPAC1L (both EC) and the ovarian carcinoma cell line OVMz were cultivated in RPMI-1640 medium (PAA Laboratories, Pasching, Austria). Media were supplemented with 10% fetal bovine serum.

Antibodies to the ectodomain of L1CAM (monoclonal antibody (mAb) L1-11A, a subclone of UJ127.11) were described before [30];[31]. The antibodies for detection in Western blot against GAPDH and p53 (DO-1) were from SantaCruz Biotechnology (Heidelberg, Germany). 5′-AzaC, TSA and Nutlin-3a were obtained for Sigma-Aldrich (St. Louis, USA) and dissolved in serum free medium or DMSO.

### Treatment of cells and biochemical analysis

Cells were seeded in 6-well plates and treated for 5 days with 5′-AzaC or for 24h with TSA. The treatment with Nutlin-3a was for 48h. Treated cells were lysed for 15 min at 4°C in 1x NuPage LDS sample buffer (LifeTech, Carlsbad, CA) and sonified. After centrifugation at lO.OOOxg for 10min at 4°C, supernatant was collected and protein concentrations were determined with a commercial protein assay (Pierce, BCA protein assay, Thermo Scientific, Waltham, USA). 50ug of protein per lane was separated by 10% SDS-polyacrylamide gel electrophoresis. The SDS gel electrophoresis and western blotting was performed as described before [15].

### FACS analysis

The cells were stained with mAb L1 -11A conjugated with Alexa488 against L1CAM as described before [30]. An isotype-matched irrelevant mAb was used as background control. Stained cells were analysed with a FACS Canto II, using FlowJo software (Becton-Dickinson, Heidelberg, Germany).

### Mimic and miRNA-inhibitor transfection

24h before the treatment 1 × 10^5^ cells were seeded per 12-well. The transfection was performed with DharmaFECT 1 reagents (Thermo Scientific, USA) following the manufacturer's protocol. For each well the final oligonucleotide concentration was 100 nM. After transfection cells were cultivated for 72h and then used for the isolation of RNA and small RNAs. The oligonucleotides were from Dharmacon Thermo Scientific, including mimic RNA, mimic control, miRNA hairpin inhibitor and inhibitor control. (miRIDIAN microRNA hsa-mir-34a mimic, miRIDIAN microRNA mimic negative control, miRIDIAN microRNA hairpin inhibitor and inhibitor control). The mimic RNA and hairpin inhibitor was specific for hsa-miR-34a-5p.

### Plasmid transfection

Transient transfection of HEC1B cells was done using FuGene (Roche, Switzerland). 24h before treatment 1 × 10^5^ cells were seeded per 12-well. For luciferase assay cells were transfected with 0.5 ug of the corresponding renilla-luciferase-UTR construct (in pLightSwitch vector), 0.83 ug miRNA expression vector (in pCMV-MIR) and 10 ng firefly-luciferase-vector (in pGL3-vector with SV40 promoter). For the overexpression of miR-34a, cells were transfected with 1.33 ug expression vector. Cells were harvested after 48 h of transfection and subjected to further analysis.

### 3′UTR-Luciferase assay

The Renilla-L1CAM-3′UTR plasmid was purchased from SwitchGear Genomics (Menlo Park, CA, USA) and combined with the dual luciferase assay from Promega (Madison, WI, USA). Cells were lysed 48 h after transfection in a 12-well plate, and the renilla and firefly luciferase activity was detected by the Promega dual luciferase assay kit. Co-transfection with firefly luciferase served as a transfection efficiency control and the renilla luciferase signal was normalized to the firefly signal. After the measurement the activities was calculated as a quotient of renilla to firefly signal and were set in relation to the pCMV vector control.

### Quantitative RT-PCR

To check the mRNA level 10ng of cDNA was analyzed in triplicates. The PCR reactions were performed with the SYBRgreen Master Mix from Applied Biosystems in an ABI 7300 analyzer. Specific primers for mRNA level detection were determined with the online tool “Primer Express” (Applied Biosystems, Foster City, CA). Primers were produced by Sigma-Aldrich (St. Louis, USA) and used in a concentration of 300 μM. The sequences for the primers were published before [15].

For detection of the miRNAs with SYBRgreen, 2μl of the corresponding 1:10 diluted reverse transcription product was used. The stem loop reverse transcription of the miR-34a and RNU48 was adopted from the publication of Chen et al. [32]. Both reverse transcription products were used to perform a qRT-PCR with specific primers for miR-34a and RNU48. Primer sequences can be provided on request.

### Expression analyses of single miRNAs with TaqMan miRNA assays

TaqMan microRNA assay specific for miR-34a (Applied Biosystems, Assay ID 000426) was used to detect and quantify mature miR-34a. The assays were performed in accordance with manufacturer's instructions (Applied Biosystems, Carlsband, CA). miRNA expression was normalized to RNU48 (Applied Biosystems, Assay ID 001093) using the 2^ΔΔCt^ method.

### miRNA screening

Genome-wide miRNA expression was analysed by quantitative PCR using the TaqMan Array Human MicroRNA Cards Set v3.0 following the manufacturer's specifications (Applied Biosystems, Foster City, CA). Briefly, 30 ng total RNA was reverse transcribed with each of the Megaplex RT Primers separately (Human Pool

A v2.1 and Pool B v3.0) using the TaqMan MicroRNA Reverse Transcription Kit. Reverse transcription products were pre-amplified using the PreAmp Mastermix and Megaplex PreAmp primers (Human Pool A v2.1 and Pool B v3.0). Diluted pre-amplification products and TaqMan Universal PCR Master Mix were dispensed into the MicroRNA Cards and quantitative PCR was performed on the 7900 HT Fast Real-time PCR System (Applied Biosystems). CT values were determined using a baseline defined from cycle 3 to 15 and a threshold of 0.2. Raw CT values were median normalized for A and B Cards separately. Differentially expressed miRNAs between untreated and 5′-AzaC treated cells were identified by a paired t-test including 398 miRNAs that were measurable in all 4 samples.

### Construction of miRNA Expression Plasmids

All miRNA sequences were cloned into the *SgfI* and *Mlu I* restriction sites of the pCMV-MIR vector (OriGene Technologies, Rockville MD, USA). The miRNAs were amplified from genomic DNA of tumor cell lines and the primers for cloning were located approximately 300 bp upstream and downstream of the mature target miRNA-sequence. A 6 bp linker sequence and the restriction enzyme sequence were added to the target primer sequences of the forward and reverse primer. The forward primers contains the *Sgf I* and the reverse primers contains the *Mlu I* sides. Sequences of the all primers are available on request. All PCR fragments were cloned into the pCMV-MIR vector and sequenced for control.

### Plasmid mutagenesis

Site directed mutagenesis within the L1CAM-3′UTR renilla fusion plasmid were carried out using a mutagenesis kit (QuikChange II Site-Directed Mutagenesis Kit) following the manufacturer's instructions. For the exchange of the nucleic acids within the miR-34a binding sites the specific primers were used. The sequences of the mutagenesis primers can be provided on request.

### Migration assay

For the migration assay the cells were transfected as described above. Cells were cultured in serum free medium for 24h. After the starving 5 × l0^4^ HEC1B cells in 100 μ1 were seeded in a CIM 16 well E-plate. The continuous migration of the cells was documented and analysed in a time-resolved manner with the xCELLigence RTCA MP instrument and the RTCA Software 1.2 (Roche diagnostics, Mannheim, Germany) to measure the CI values. The migration was measured over a time period of 48h. During the measurement the lower chambers contained full growth medium as chemoattractant and the upper chambers medium without FCS.

### Screening for p53 function

A functional yeast-based assay was employed to analyse inactivating p53 mutations in EC cell lines as previously described [33]. In summary, mRNA was isolated and reverse transcribed, then p53 was amplified by PCR, and co-transfected into saccharomyces cervisiae with a linearized yeast expression vector carrying the 5′ and 3′ ends of the p53 open reading frame. Expression of transcriptionally active p53, which activates transcription of the yeast ADE2 gene, results in white colonies, whereas mutant alleles often lack transcriptional activity and result in smaller, red colonies. DNA from at least two red colonies was sequenced to characterize the p53 mutations.

### Microdissection of tumor tissue and RNA isolation

ECs were collected at the Department of Gynecology and Obstetrics, Medical University of Innsbruck. This project was approved by the local ethics committee (University of Innsbruck, UN3801; reference number: 282/4.12).

In total, we analyzed 9 endometrioid ECs (8 endometrioid ECs with areas of squamous differentiation), 7 clear cell ECs, 10 papillary serous ECs and 4 normal endometrial tissues. Immunohistochemistry for L1CAM was conducted as described elsewhere in detail [10]. RNA from FFPE punch biopsies was extracted using the RecoverAll™ Total Nucleic Acid Isolation Kit for FFPE (AM1975; Life Technologies, Vienna, Austria).

## References

[1] Hanahan D, Weinberg RA (2000). The hallmarks of cancer. Cell.

[2] Hanahan D, Weinberg RA (2011). Hallmarks of cancer: the next generation. Cell.

[3] Pfeifer M, Schirmer U, Geismann C, Schafer H, Sebens S, Altevogt P (2010). L1CAM expression in endometrial carcinomas is regulated by usage of two different promoter regions. BMC Mol Biol.

[4] Kiefel H, Bondong S, Pfeifer M, Schirmer U, Erbe-Hoffmann N, Schafer H, Sebens S, Altevogt P (2012). EMT-associated up-regulation of L1CAM provides insights into L1CAM-mediated integrin signalling and NF-kappaB activation. Carcinogenesis.

[5] Voulgari A, Pintzas A (2009). Epithelial-mesenchymal transition in cancer metastasis: mechanisms, markers and strategies to overcome drug resistance in the clinic. Biochimica et biophysica acta.

[6] Thiery JP (2002). Epithelial-mesenchymal transitions in tumour progression. Nat Rev Cancer.

[7] Schafer MK, Altevogt P (2010). L1CAM malfunction in the nervous system and human carcinomas. Cell Mol Life Sci.

[8] Kiefel H, Bondong S, Hazin J, Ridinger J, Schirmer U, Redle S, Altevogt P (2012). L1CAM: a major driver for tumor cell invasion and motility. Cell adhesion & migration.

[9] Gavert N, Ben-Shmuel A, Raveh S, Ben-Ze'ev A (2008). L1-CAM in cancerous tissues. Expert Opin Biol Ther.

[10] Huszar M, Pfeifer M, Schirmer U, Kiefel H, Konecny GE, Ben-Arie A, Edler L, Munch M, Muller-Holzner E, Jerabek-Klestil S, Abdel-Azim S, Marth C, Zeimet AG, Altevogt P, Fogel M (2010). Up-regulation of L1CAM is linked to loss of hormone receptors and E-cadherin in aggressive subtypes of endometrial carcinomas. J Pathol.

[11] Zeimet AG, Reimer D, Huszar M, Winterhoff B, Puistola U, Azim SA, Muller-Holzner E, Ben-Arie A, van Kempen LC, Petru E, Jahn S, Geels YP, Massuger LF, Amant F, Polterauer S, Lappi-Blanco E (2013). L1CAM in Early-Stage Type I Endometrial Cancer: Results of a Large Multicenter Evaluation. Journal of the National Cancer Institute.

[12] Geismann C, Arlt A, Bauer I, Pfeifer M, Schirmer U, Altevogt P, Muerkoster SS, Schafer H (2011). Binding of the transcription factor Slug to the L1CAM promoter is essential for transforming growth factor-betal (TGF-beta)-induced L1CAM expression in human pancreatic ductal adenocarcinoma cells. Int J Oncol.

[13] Kuwajima A, Iwashita J, Murata J, Abe T (2007). The histone deacetylase inhibitor butyrate inhibits melanoma cell invasion of Matrigel. Anticancer Res.

[14] Kato K, Maesawa C, Itabashi T, Fujisawa K, Otsuka K, Kanno S, Tada H, Tatemichi Y, Kotani K, Oikawa H, Sugai T, Wakabayashi G, Masuda T (2009). DNA hypomethylation at the CpG island is involved in aberrant expression of the L1 cell adhesion molecule gene in colorectal cancer. Int J Oncol.

[15] Schirmer U, Fiegl H, Pfeifer M, Zeimet AG, Müller-Holzner E, Bode PK, Tischler V, Altevogt P (2013). Epigenic regulation of L1CAM in endometrial carcinoma: comparison to cancer-testis (CT-X) antigens. BMC Cancer.

[16] Croce CM (2009). Causes and consequences of microRNA dysregulation in cancer. Nat Rev Genet.

[17] Iorio MV, Croce CM (2012). microRNA involvement in human cancer. Carcinogenesis.

[18] Hunten S, Siemens H, Kaller M, Hermeking H (2013). The p53/microRNA network in cancer: experimental and bio informatics approaches. Advances in experimental medicine and biology.

[19] Vassilev LT, Vu BT, Graves B, Carvajal D, Podlaski F, Filipovic Z, Kong N, Kammlott U, Lukacs C, Klein C, Fotouhi N, Liu EA (2004). In vivo activation of the p53 pathway by small-molecule antagonists of MDM2. Science.

[20] Kumamoto K, Spillare EA, Fujita K, Horikawa I, Yamashita T, Appella E, Nagashima M, Takenoshita S, Yokota J, Harris CC (2008). Nutlin-3a activates p53 to both down-regulate inhibitor of growth 2 and up-regulate mir-34a, mir-34b, and mir-34c expression, and induce senescence. Cancer Res.

[21] Hermeking H (2012). MicroRNAs in the p53 network: micromanagement of tumour suppression. Nat Rev Cancer.

[22] Siemens H, Jackstadt R, Hunten S, Kaller M, Menssen A, Gotz U, Hermeking H (2011). miR-34 and SNAIL form a double-negative feedback loop to regulate epithelialmesenchymal transitions. Cell cycle.

[23] Hwang CI, Matoso A, Corney DC, Flesken-Nikitin A, Korner S, Wang W, Boccaccio C, Thorgeirsson SS, Comoglio PM, Hermeking H, Nikitin AY (2011). Wild-type p53 controls cell motility and invasion by dual regulation of MET expression. Proceedings of the National Academy of Sciences of the United States of America.

[24] Kim NH, Kim HS, Li XY, Lee I, Choi HS, Kang SE, Cha SY, Ryu JK, Yoon D, Fearon ER, Rowe RG, Lee S, Maher CA, Weiss SJ, Yook JI (2011). A p53/miRNA-34 axis regulates Snaill-dependent cancer cell epithelial-mesenchymal transition. J Cell Biol.

[25] Reimer D, Hubalek M, Kiefel H, Riedle S, Skvortsov S, Erdel M, Hofstetter G, Concin N, Fiegl H, Muller-Holzner E, Marth C, Altevogt P, Zeimet AG (2011). Regulation of transcription factor E2F3a and its clinical relevance in ovarian cancer. Oncogene.

[26] Garofalo M, Jeon YJ, Nuovo GJ, Middleton J, Secchiero P, Joshi P, Alder H, Nazaryan N, Di Leva G, Romano G, Crawford M, Nana-Sinkam P, Croce CM (2013). MiR-34a/c-Dependent PDGFR-alpha/beta Downregulation Inhibits Tumorigenesis and Enhances TRAIL-Induced Apoptosis inLung Cancer. PloS one.

[27] Siemens H, Jackstadt R, Kaller M, Hermeking H (2013). Repression of c-Kit by p53 is mediated by miR-34 and is associated with reduced chemoresistance, migration and sternness. Oncotarget.

[28] Yeramian A, Moreno-Bueno G, Dolcet X, Catasus L, Abal M, Colas E, Reventes J, Palacios J, Prat J, Matias-Guiu X (2013). Endometrial carcinoma: molecular alterations involved in tumor development and progression. Oncogene.

[29] Cancer Genome Atlas Research N, Kandoth C, Schultz N, Cherniack AD, Akbani R, Liu Y, Shen H, Robertson AG, Pashtan I, Shen R, Benz CC, Yau C, Laird PW, Ding L, Zhang W, Mills GB (2013). Integrated genomic characterization of endometrial carcinoma. Nature.

[30] Mechtersheimer S, Gutwein P, Agmon LN, Stoeck A, Oleszewski M, Riedle S, Postina R, Fahrenholz F, Fogel M, Lemmon V, Altevogt P (2001). Ectodomain shedding of L1 adhesion molecule promotes cell migration by autocrine binding to integrins. J Cell Biol.

[31] Gast D, Riedle S, Issa Y, Pfeifer M, Beckhove P, Sanderson MP, Arlt M, Moldenhauer G, Fogel M, Kruger A, Altevogt P (2008). The cytoplasmic part of L1-CAM controls growth and gene expression in human tumors that is reversed by therapeutic antibodies. Oncogene.

[32] Chen C, Ridzon DA, Broomer AJ, Zhou Z, Lee DH, Nguyen JT, Barbisin M, Xu NL, Mahuvakar VR, Andersen MR, Lao KQ, Livak KJ, Guegler KJ (2005). Real-time quantification of microRNAs by stem-loop RT-PCR. Nucleic Acids Res.

[33] Deissler H, Kafka A, Schuster E, Sauer G, Kreienberg R, Zeillinger R (2004). Spectrum of p53 mutations in biopsies from breast cancer patients selected for preoperative chemotherapy analysed by the functional yeast assay to predict therapeutic response. Oncology reports.

